# Emerging advanced approaches for liquid biopsy: *in situ* nucleic acid assays of extracellular vesicles

**DOI:** 10.7150/thno.102437

**Published:** 2024-10-28

**Authors:** Dongli Wang, Ye Shen, Hui Qian, Jiajia Jiang, Wenrong Xu

**Affiliations:** 1 Aoyang Institute of Cancer, Affiliated Aoyang Hospital of Jiangsu University, 279 Jingang Road, Suzhou Jiangsu 215600, China.; 2 Zhenjiang Key Laboratory of High Technology Research on Exosomes Foundation and Transformation Application, Jiangsu Key Laboratory of Medical Science and Laboratory Medicine, School of Medicine, Jiangsu University, Zhenjiang Jiangsu 212013, China.

**Keywords:** extracellular vesicles, cancer, liquid biopsy, detection, diagnosis

## Abstract

Extracellular vesicles (EVs) have emerged as valuable biomarkers in liquid biopsies owing to their stability, accessibility, and ability to encapsulate nucleic acids. The majority of existing methodologies for detecting EV nucleic acid biomarkers require the lysis of EVs to extract DNA or RNA. This process is labor-intensive and may lead to the loss and degradation of nucleic acids. However, the emerging field of *in situ* EV assays offers innovative tools for liquid biopsy, facilitating direct profiling of nucleic acids within intact EVs and reducing sample handling procedures. This review focuses on the promising and innovative field of *in situ* EV nucleic acid analysis. It examines the translational potential of *in situ* EV nucleic acid analysis in liquid biopsies from detection strategies, diagnostic applications, and diagnostic aids for single EV analysis and machine learning techniques. We highlight the innovative approach of *in situ* EV nucleic acid assays and provide novel insights into advancing liquid biopsy technology. This approach shows a promising avenue for improving EV-based cancer diagnosis and guiding personalized treatment with minimal invasiveness.

## Introduction

Cancer is one of the leading causes of death in humans 1. Traditional tumor diagnosis methods, such as histopathological biopsy, are invasive and can pose risks and financial burdens to patients. As a result, clinics are turning to liquid biopsy, which is a non-invasive or minimally invasive, convenient, rapid, and cost-effective alternative 2. Extracellular vesicles (EVs) are particles released by all cell types, characterized by their lipid bilayer membranes. These vesicles carry a diverse range of biologically active substances, including proteins, lipids, nucleic acids, and metabolites, which provide crucial insights into the physiological status of diseases and play a significant role in mediating cellular communication 3-6. Research has shown that EVs contribute to tumor development by promoting cancer cell proliferation, metastasis, angiogenesis, drug resistance, and immune evasion 7, 8. Furthermore, EVs are abundant and stable in body fluids, making them promising biomarkers for cancer diagnosis, treatment, and monitoring 9.

EV-based liquid biopsy presents a promising approach for diagnosing tumors in clinical patients. However, accurately and rapidly detecting these biomarkers in bodily fluids is a major challenge in clinical practice. The quantitative analysis of EV nucleic acids is usually conducted using traditional methods such as quantitative real-time PCR (qRT-PCR), which is cumbersome and low sensitive 10. Recently, a variety of innovative techniques for detecting EV nucleic acids have started to emerge. These biosensors eliminate the need for conventional cDNA synthesis and enzymatic amplification, offering advantages in sensitivity, specificity, and ease of operation over qRT-PCR 11. For instance, Lei Zheng's team have developed an electrochemical biosensor that employs localized DNA tetrahedron-assisted catalytic hairpin assembly for efficient and specific detection of plasma-derived EV miRNAs in gastric cancer 12. Kang *et al.* have designed a SERS-based sensor using a gold octahedra array as a substrate for sensitive detection of breast cancer-derived exosomal let-7a 13. A peptide nucleic acid-functionalized nanochannel biosensor has shown good agreement with qRT-PCR in detecting exosomal miR-10b for early diagnosis of pancreatic cancer 14. An SPR biosensor using Au-on-Ag heterostructure and DNA tetrahedral framework enables ultra-sensitive detection of multiple miRNAs of exosomal origin from NSCLC 15. Nevertheless, due to the protective nature of nucleic acids in EVs by lipid bilayers, the above EV nucleic acid assays still require labor-intensive processes of EV lysis and nucleic acid extraction, and they are susceptible to nucleic acid loss and degradation 16. Consequently, direct analysis of nucleic acids from intact EVs is crucial for advancing liquid biopsy techniques. *In situ* EV nucleic acid detection is a technique that directly analyzes nucleic acids within EVs while preserving EV integrity. It eliminates the need for EV lysis and nucleic acid extraction, thereby streamlining sample handling procedures.

Over the past few years, many reviews have been published on EV liquid biopsy techniques, including fluorescence, electrical or electrochemical, surface plasmon resonance (SPR), surface-enhanced Raman scattering (SERS), colorimetry, microfluidics, and various nanomaterial biosensors 17-22. Nevertheless, these reviews have mainly focused on detection methods and protein analyses, with only a few specifically addressing the detection of EV nucleic acid biomarkers. Furthermore, the development of *in situ* detection techniques for directly analyzing nucleic acid from intact EVs is still in its early stages, and a comprehensive investigation of their potential for application in liquid biopsies has not been documented yet. Notably, *in situ* EV nucleic acid analysis emphasizes the importance of probes being able to directly penetrate EVs for effective nucleic acid detection, which is not essential for accurately identifying EV subtypes. Single EV analysis has been developed to elucidate EV heterogeneity, with methods such as fluorescent labelling for identifying nucleic acids in single intact EVs playing a significant role in reflecting disease progression and improving the timeliness of disease diagnosis 23, 24. This serves as a vital complement to *in situ* EV nucleic acid assays. Nonetheless, there is currently a lack of comprehensive summaries on single EV analysis techniques from the perspective of *in situ* EV nucleic acid detection. As a result, we have intentionally included a discussion on this topic in the manuscript to advance the development of *in situ* EV nucleic acid assays.

This review begins with an introduction to the biological role of EVs in tumors and their potential as biomarkers for tumor diagnosis. Subsequently, it offers a comprehensive overview of current strategies for *in situ* analysis of EV nucleic acids. These strategies encompass fluorescence detection employing various probe structures, microfluidics chips, and SERS detection. Furthermore, the review explores recent advancements in single EV analysis and machine learning-assisted *in situ* EV nucleic acid detection. Additionally, it summarizes the application of *in situ* EV nucleic acid analysis in liquid biopsies. Finally, the review delves into the prospects and existing challenges related to the implementation of *in situ* EV nucleic acid assays in liquid biopsies, and provides potential recommendations for enhancing their transition to clinical practice. In conclusion, this review aims to promote the progress of EV-based liquid biopsy techniques, offering valuable guidance and insights for the diagnosis and treatment of cancer.

## Extracellular vesicles

### Characteristics and biological roles of EVs

**Figure 1:**
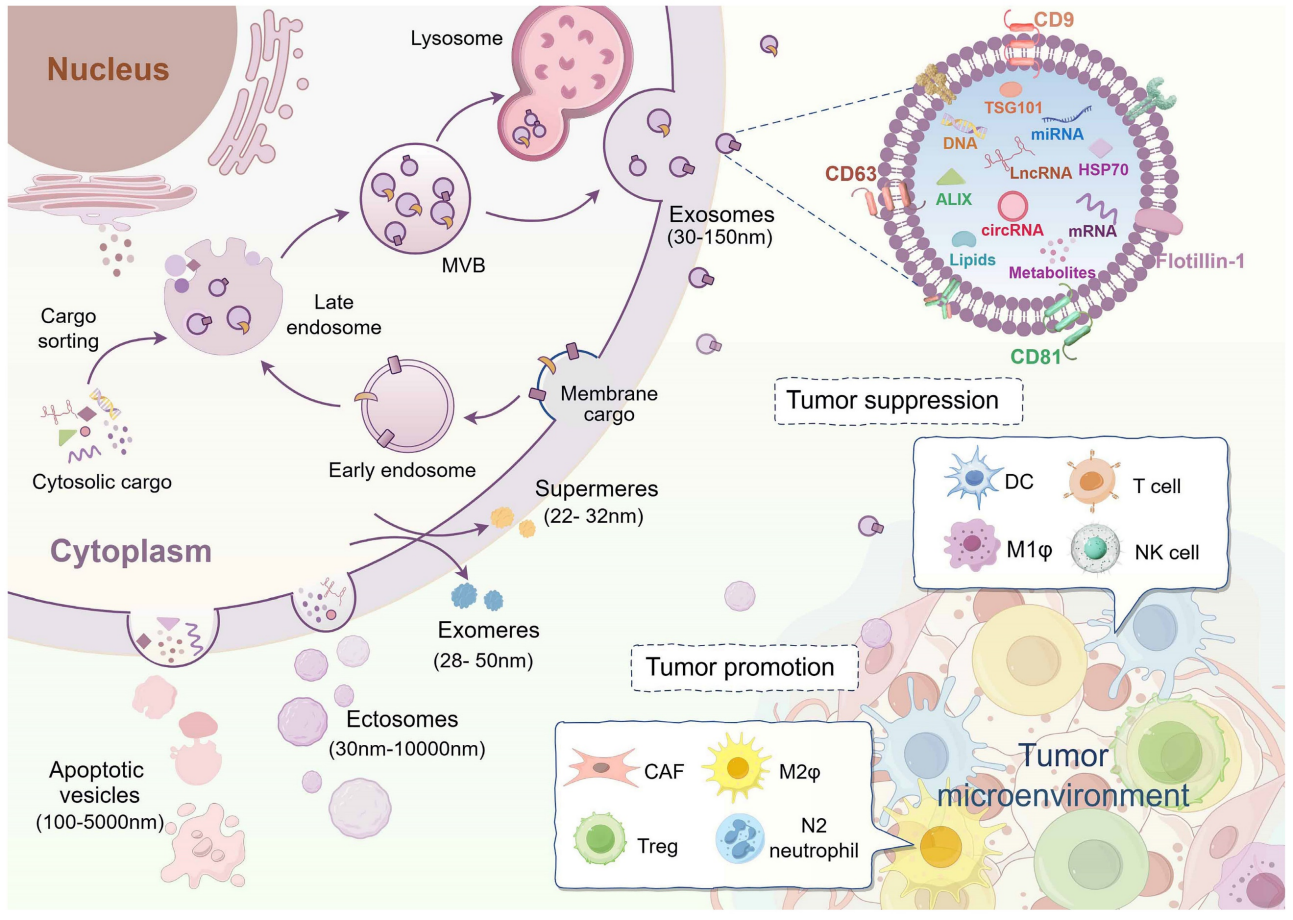
** Biogenesis of extracellular vesicles and their roles in the tumor microenvironment.** Extracellular vesicles (EVs) with lipid bilayers consist of various isoforms. Among these subtypes, exosomes, originating from the endosomal system, are the most extensively researched category of small extracellular vesicles (sEV). Ectosomes, derived from the cytoplasmic membrane, exhibit a range of sizes. Apoptotic vesicles are generated by cells undergoing apoptosis. Exomeres and supermeres are the latest discovery of non-membranous nanoparticles that can be secreted into the extracellular compartment. Although they are much smaller in size, the process of their formation remains unclear. EVs represented by exosomes contain a variety of cargoes, including nucleic acids, proteins, lipids, and metabolites. EVs serve as critical mediators of intercellular communication and assume a biphasic role within the tumor microenvironment. Tumor-associated fibroblasts (CAF), M2-like macrophages (M2ф), regulatory T cells (Tregs), and N2 neutrophils-derived EVs predominantly contribute to the promotion of tumor progression. By contrast, EVs from dendritic cells (DCs), T cells, M1-like macrophages (M1ф), and natural killer (NK) cells function as tumor-suppressive effects. (Created with Figdraw.com).

EVs are categorized into distinct subtypes based on variations in size and origin. Specifically, vesicles with a diameter of smaller than 200 nm are often referred to as small extracellular vesicles (sEV). Depending on how they are formed, EVs can be classified as exosomes (30-150 nm) originating from the endosomal system and ectosomes (30-10,000 nm) originating from cytoplasmic membrane. Exosomes, a subset of small extracellular vesicles (sEVs), form through fusion with the cytoplasmic membrane following the inward budding of late endosomes to create multivesicular bodies. Ectosomes exhibit a diverse range of sizes, including various EVs found in cell-specific conditions, such as apoptotic bodies or vesicles (100-5000 nm). They are produced through the direct secretion from apoptotic cell membranes or the cellular debris following cell death 3, 5, 25. Notably, in addition to the membrane vesicles mentioned above, recent research has identified smaller-sized (<50 nm) non-membrane nanoparticles, like exomeres and supermeres, although the mechanism underlying their formation remains unknown (**Figure 1**) 26, 27. EVs exhibit heterogeneity in terms of size, quantity, composition, cellular sources, and physiological roles 28. Factors contributing to variations in EV sizes include differences in cytoplasmic membrane invagination, membrane budding, and isolation methodologies. The diverse cellular sources and differences in size directly impact the content and composition of EVs. Since EV-carried cargoes are derived from parental cells, EVs from different cellular sources exert different biological functions on target cells. For example, EVs from stem cells are involved in disease repair mechanisms, while EVs from tumor cells contribute to cancer progression 29-35.

In recent years, EVs have emerged as crucial players in intercellular communication within the tumor microenvironment, exhibiting both promoting and inhibitory effects on cancer progression (**Figure 1**) 36. EVs released by cells in the cancer microenvironment have been shown to enhance tumor growth, angiogenesis, metastasis, drug resistance, and immune evasion, thereby contributing to tumor progression. For example, EVs from M2-like macrophages (M2ф) stimulate angiogenesis and facilitate the growth of pancreatic ductal adenocarcinoma (PDAC) by targeting E2F2 37. Similarly, nicotine-activated N2-neutrophils secrete EV miR-4466 to promote stemness and metabolism of brain metastatic tumor cells in lung cancer 38. Cancer-associated fibroblast (CAF)-derived EVs inhibit ferroptosis via the miR-432-5p/CHAC1 axis, thereby increasing resistance to docetaxel in prostate cancer 39. Additionally, breast cancer-derived EV lncRNA SNHG16 induces amplification of CD73+γδ1 regulatory T cells (Tregs) and consequently exerts an immunosuppressive effect on tumors 40.

Currently, tumor EVs with anti-cancer properties predominantly originate from immune cells, and researchers are focusing on cancer immunotherapy by modifying or enhancing the functionality of these immune cells. Dendritic cells (DCs), which are highly efficient antigen-presenting cells, release a significant quantity of EVs that trigger strong anti-cancer responses. For example, dendritic cell-derived EVs enriched with RAE-1γ can stimulate NK cells and T cells to eliminate chronic myeloid leukemia (CML) cells, regardless of the presence of the T315I mutation, resulting in effective anti-cancer outcomes 41. Other sources of EVs capable of exerting anti-cancer effects include T cells 42, NK cells 43, and M1-like macrophages (M1ф) 44.

EVs, with their double-layer membrane structure, serve as optimal cargo transportation carriers, protecting cargo from degradation. Moreover, EVs exhibit excellent biocompatibility and prolonged circulation, making them suitable for delivering therapeutic drugs or molecules to specific cells for disease treatment 45. For instance, *in vivo* administration of EV-mediated si-ciRS-122 reversed oxaliplatin resistance and suppressed the proliferation of colorectal cancer (CRC) tumors 34. Small extracellular vesicles (sEVs) derived from human umbilical cord mesenchymal stem cells enhanced retinopathy recovery in diabetic mice by loading miR-5068 and miR-10228 through electroporation 29. Engineered EVs, such as those utilizing the tumor-targeting peptide RGD, have emerged as a promising platform for targeted drug delivery in tumor therapies. By encapsulating circDIDO1, these engineered EVs effectively suppressed the growth of gastric tumors in nude mice 46. Neutrophil-derived EVs engineered with superparamagnetic iron oxide nanoparticles (SPIONs) improved tumor localization accuracy. Similarly, nano-sized vesicles derived from neutrophils (NNVs), functionalized with SPIONs and encapsulated with DOX, showed targeted accumulation at the tumor site under an external magnetic field. This resulted in robust suppression of gastric cancer (GC) progression 47. In a comprehensive review, Zhang *et al.* provided detailed insights into the strategies and methodologies involved in engineering EVs 48.

### EV-based liquid biopsy of cancer

**Figure 2:**
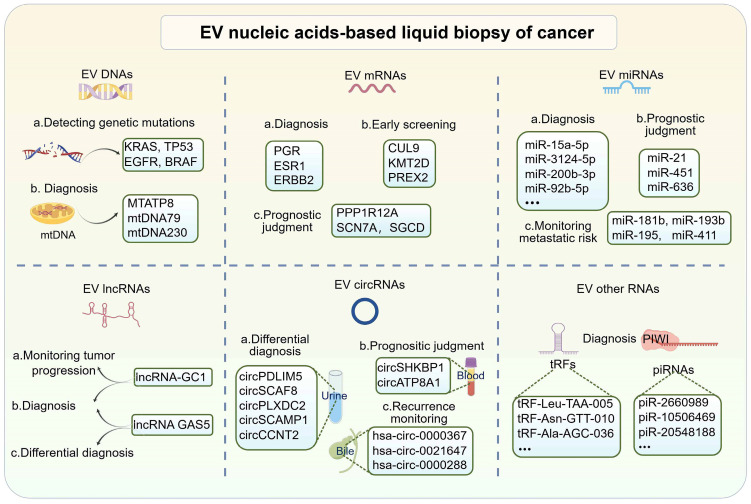
**Significance of EV nucleic acid biomarkers in liquid biopsy.** EV nucleic acid-based liquid biopsies for cancer have demonstrated substantial potential. DNA analysis in EVs has primarily targeted mutation detection in tumors, highlighting mitochondrial DNA (mtDNA) as a promising innovation for liquid biopsy biomarkers. A variety of RNA types within EVs have been identified as biomarker potential. These RNA biomarkers, such as mRNAs, miRNAs, lncRNAs, circRNAs, and others, have exhibited promising utility in tumor diagnosis, identification, screening, prognosis, and recurrence monitoring. (Created with Figdraw.com).

In recent years, EV-based liquid biopsies have gained traction in clinical diagnostic research. Compared to circulating tumor cells (CTCs), EVs offer advantages such as smaller size, wider distribution, and more accessible enrichment in bodily fluids. Additionally, the unique lipid bilayer structure of EVs protects their cargo from degradation, distinguishing them from circulating tumor DNA (ctDNA) or cell-free RNA (cfRNA) and making them ideal components for liquid biopsies 49. EVs contain abundant nucleic acid components, including DNA, mRNA, miRNA, lncRNA, circRNA, and others. This section provides an overview of prevalent nucleic acid biomarkers in EVs (**Figure 2**).

#### EV-derived DNAs

Currently, DNA liquid biopsies primarily focus on ctDNA. The study of EV DNAs, despite its data, biological stability, and clinical relevance, has been limited due to its minimal content of large DNA fragments 50. DNAs from EVs primarily serve as a diagnostic biomarker for identifying tumor gene mutations in clinical settings. Studies have demonstrated that identifying mutations in plasma EV-derived KRAS can predict colorectal cancer (CRC) and pancreatic ductal adenocarcinoma (PDAC) 51, 52. Similarly, mutations in EV TP53 have been linked to hepatocellular carcinoma (HCC) prognosis 53. Due to the low concentration of EV DNAs, digital PCR is commonly used to enhance the sensitivity of detecting these rare DNA mutations. For instance, Choi *et al.* achieved a higher detection rate of KRAS mutations in EVs from CRC patients compared to cfDNA using digital PCR, with a sensitivity of 76% and specificity of 100% 52. Additionally, EGFR and BRAF mutations in EVs are implicated in cancer diagnosis 54, 55. Mitochondrial DNA (mtDNA) is another non-invasive molecule with diagnostic significance in cancer, and it can be detected in circulating bodily fluids due to its high copy number 56. Recent studies have demonstrated the biomarker potential of mtDNA in EVs. Lou *et al.* identified elevated levels of specific mtDNA fragments (mtDNA79, mtDNA230, and MTATP8) in plasma EVs from non-small cell lung cancer (NSCLC). These mtDNA fragments exhibited a strong association with more aggressive NSCLC traits, including larger tumors, advanced stage, lymph node metastasis, and distant metastasis, indicating significant potential for NSCLC diagnosis 57.

#### EV-derived mRNAs

mRNAs can be transferred between cells through EVs. mRNAs from EVs have shown potential as a liquid biopsy method. Studies have demonstrated that EV mRNAs in blood and urine can be used to diagnose urinary tract diseases. For example, a recent study on breast cancer found that a combination of specific mRNA signatures (PGR, ESR1, ERBB2, and GAPDH) derived from EVs significantly enhanced the accuracy of breast cancer diagnosis when used in conjunction with multiplexed detection using ddPCR and a machine-learning algorithm (AUC=0.95). This approach has the potential to expedite the discovery of low-abundance nucleic acid biomarkers discovery in EVs, thereby advancing the prospect of early cancer screening based on EV mRNA profile 58. He *et al.* identified five EV mRNAs (CUL9, KMT2D, PBRM1, PREX2, and SETD2) as novel potential biomarkers for clear cell renal cell carcinoma (ccRCC). Among these, KMT2D and PREX2 showed an early diagnosis of ccRCC (AUC=0.83), while CUL9, KMT2D, and PREX2 could differentiate between patients with ccRCC and those with benign renal masses (AUC=0.81) 59. Moreover, EV mRNAs excel in prognostic assessment. A study identified a prognostic EV mRNA signature (PPP1R12A, SCN7A, and SGCD) through transcriptomic analysis of plasma EV from pancreatic cancer patients. This signature correlated with a decreased overall survival rate (*p* = 0.014) in high-risk PDAC patients, establishing it as an independent prognostic biomarker for PDAC 60.

#### EV-derived miRNAs

MicroRNAs (miRNAs) are short non-coding RNAs (~22 bases) that regulate protein-coding genes at the post-transcriptional level 61. They play a crucial role in tumor development and liquid biopsy by destabilizing target mRNAs and suppressing their translation 62. Many studies have investigated the potential of EV miRNAs as tumor biomarkers. For instance, elevated levels of plasma-derived EV miR-15a-5p, miR-106b-5p, and miR-107 have been observed in endometrial cancer (EC). Notably, miR-15a-5p exhibited an AUC value of 0.813 in distinguishing between stage I EC patients and healthy donors. Combining miR-15a-5p with CEA and CA125 improved the AUC value of 0.899 63. In another study, up-regulation of serum-derived EV miRNAs (miR-3565, miR-3124-5p, miR-200b-3p, miR-6515, miR-3126-3p, and miR-9-5p) were found in patients with small-cell lung cancer (SCLC), while miR-92b-5p showed down-regulation. The combined measurement of three miRNAs (miR-200b-3p, miR-3124-5p, and miR-92b-5p) significantly improved early diagnostic efficiency (AUC = 0.93) 64. Additionally, Sun *et al.* established a metastatic risk score model using miR-21, miR-451, and miR-636 extracted from urinary EVs in prostate cancer. This model accurately predicted metastasis with an AUC value of 0.925, surpassing the predictive ability of the preoperative PSA levels or clinical Gleason scores. It shows promise as a noninvasive biomarker for predicting prognosis in PCa patients 65. Moreover, four EV miRNAs (miR-181b, miR-193b, miR-195, and miR-411) have shown robustness in detecting lymph node metastasis (LNM) in CRC patients. A risk stratification model was established by incorporating key pathologic features, which reduced the false-positive rate of LNM to 76% without missing any actual LNM-positive patients 66. Thus, EV miRNA can be used to monitor tumor progression and predict risk stratification for metastasis.

#### EV-derived lncRNAs

Long non-coding RNAs (LncRNAs) are nucleotide sequences longer than 200 nucleotides that do not encode proteins. They play crucial roles in regulating gene expression at the transcriptional and post-transcriptional levels, contributing to cancer pathogenesis 67. EV-derived lncRNAs hold promise as biomarkers for tumors 68. For example, circulating EV lncRNA-GC1 has been shown to diagnose early GC and monitor disease progression. Compared to CEA, CA72-4, and CA19-9, lncRNA-GC1 showed higher diagnostic accuracy for GC with an AUC of 0.89, along with sufficient specificity (84.97%) and sensitivity (84.77%). Furthermore, the level of EV lncRNA-GC1 was significantly correlated with GC progression 69. Additionally, lncRNA-GC1 levels in circulating EVs showed strong agreement (>50% reductions) with imaging response (Cohen's κ, 0.704), serving as a biomarker for evaluating the efficacy of neoadjuvant chemotherapy (neoCT) and predicting survival outcomes in GC patients receiving neoCT 70. Moreover, the tumor-derived EV lncRNA GAS5 can serve as an early diagnostic biomarker for NSCLC. EV-GAS5 was down-regulated in NSCLC patients compared to healthy controls and exhibited an AUC of 0.822 in identifying stage I tumors. When combined with CEA, the AUC value of Exo-GAS5 reached 0.929 71.

#### EV-derived circRNAs

Circular RNAs (circRNAs) are stable and abundant single-stranded, non-coding RNA molecules with covalent closed-loop structures. They play a crucial role in regulating various cellular processes in mammals, acting as miRNA sponges, protein decoys, molecular scaffolds, transcriptional regulators, and translational polypeptides 72. Additionally, circRNAs are enriched in EVs and can be transported to target cells, contributing to tumor development. The exceptional stability and tissue-specific expression patterns of EV circRNAs make them highly potential and advantageous biomarkers for liquid biopsy in cancer 73, 74. Initial studies identified over 1000 circRNAs in serum EVs that distinguish CRC patients from individuals 75. Subsequently, additional circRNA biomarkers in EVs have been discovered in different biological samples. For instance, the elevated levels of circSHKBP1 and circATP8A1 in blood-derived EVs from gastric cancer (GC) patients are associated with advanced TNM staging and unfavorable prognosis, suggesting their promise as a diagnostic and prognostic biomarker 76, 77. In urine, an extracellular vesicular circRNA classifier (Ccirc) containing circPDLIM5, circSCAF8, circPLXDC2, circSCAMP1, and circCCNT2 demonstrated the ability to detect high-grade prostate cancer (PCa) at initial biopsy, achieving an NPV of 87.50% and a sensitivity of 66.39% in the validation cohort, reducing the need for puncture biopsies by 56.34% 78. Furthermore, researchers discovered a panel of circRNAs (has-circ-0000367, has-circ-0021647, and has-circ-0000288) in serum and bile EVs for early recurrence monitoring. Both bile-derived ERS and serum-derived ERS showed significant correlations with relapse-free survival (RFS) based on the computation of the early recurrence score (ERS), achieving AUCs of 0.851 and 0.759 and accuracies of 0.720 and 0.762 for bile-ERS and serum-ERS, respectively, in the recurrence monitoring model 79.

#### EV-derived other RNAs

In addition to the extensively investigated EV nucleic acid biomarkers mentioned above, EVs from tumor liquid biopsies have also been found to contain other nucleic acids such as piRNA and tsRNA. tsRNA are small non-coding RNAs derived from tRNA molecules, specifically tRNA-derived fragments (tRFs) and tRNA-derived stress-induced RNAs (tiRNAs), which are produced through enzymatic cleavage of mature tRNAs or tRNA precursors 80. piRNAs, on the other hand, are a type of RNA associated with proteins from the PIWI branch of the argonaute family and play a crucial role in germ cell development 81. Both tiRNAs and piRNAs, as small RNA molecules, have been detected in EVs and show promise as diagnostic biomarkers. For example, a study found that certain EV-derived tRFs, tRF-Leu-TAA-005, tRF-Asn-GTT-010, tRF-Ala-AGC-036, tRF-Lys-CTT-049, and tRF-Trp-CCA-057, were significantly down-regulated in early-stage NSCLC patients compared to healthy controls. When these five tRFs were combined, their diagnostic accuracy increased, suggesting their potential as diagnostic biomarkers for NSCLC 82. Furthermore, analysis of plasma EVs from cholangiocarcinoma and gallbladder cancer patients revealed upregulation of piRNAs, including piR-2660989, piR-10506469, piR-20548188, piR-10822895, piR-has-23209, and piR-18044111, as identified through RNA sequencing. Interestingly, the postoperative analysis revealed a significant reduction in the expression of piR-10506469 and piR-20548188, suggesting their potential utility as diagnostic biomarkers for cholangiocarcinoma and gallbladder cancer 83. Overall, these findings highlight the potential of tiRNAs and piRNAs in EVs as valuable diagnostic biomarkers for various types of cancer.

Apart from those above nucleic acid-based liquid biopsies, EV nucleic acid-based multi-omics characterization has shown promise in precise tumor diagnosis. For instance, a 6-EV-RNA panel (let-7i-5p, miR-1307-3p, LZIC, SRSF6, lncFTH1-211, and lncPTMA-209) identified through EV multi-omics RNA sequencing was able to robustly identified advanced GC patients treated with fluorouracil-based neoadjuvant chemotherapy. The panel achieved 100% sensitivity, specificity, and area under the ROC curve in the training cohort after analysis using the COX regression algorithm 84. Furthermore, in-depth molecular characterization of tumor heterogeneity has proven effective in risk stratification of patients 85. The application of a robust corroborative analysis for biomarker discovery (RCABD) strategy has been proposed for identifying EV molecules, validating differential expression, modeling risk prediction, and conducting heterogenous dissection with multi-omics. A panel of 10 EV signatures (RTCA, SLC1A5, miR-324-5p, AURKA, HLA-DQB2, P2RX1, COL17A1, miR-99a-5p, LINC01055, and C3) enables effective risk stratification in breast cancer, serving as a novel and precise prognostic marker with significant CoxP value, hazard ratio, and confidence interval. This provides valuable insights into exploring the relationships between biological heterogeneity, risk stratification, and prognosis prediction in cancer 86.

Despite the proven importance of EV biomarkers in tumor progression, their potential for cancer diagnosis still faces challenges. Firstly, the complexity of biological fluids impacts the purity and yield of EV isolation, thereby influencing the efficiency of extracting nucleic acid biomarkers. Secondly, more clinical samples are needed to validate the feasibility of these biomarkers. Additionally, sensitive, stable, and user-friendly assay kits are needed to facilitate the clinical application of EV nucleic acid biomarkers.

## Emerging techniques for *in situ* nucleic acid assays of EVs

**Figure 3:**
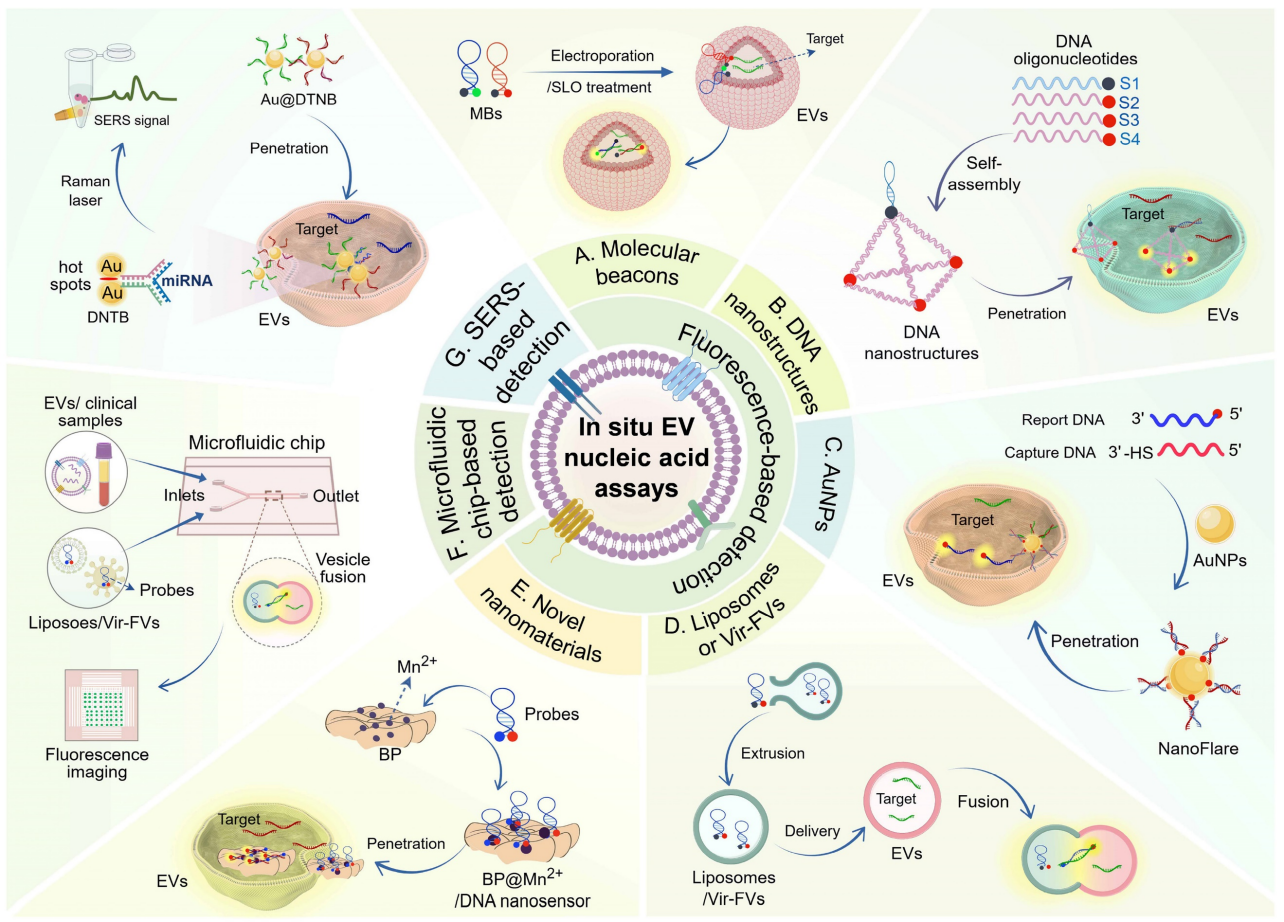
**Schematic illustration of various strategies for *in situ* EV nucleic acid assays.** Presently, there are three predominant approaches for the *in situ* detection of EV nucleic acids: fluorescence-based detection, microfluidic chips integrating fluorescence strategies, and SERS-based detection. Fluorescence detection, being the most common approach, relies on unique structural properties of various probes to enable sensitive *in situ* EV nucleic acid detection. The array of functional materials utilized in fluorescence detection includes molecular beacons (MBs) (A) for their sequence-specific recognition, DNA nanostructures (B) for their strong membrane permeability and customizable design, gold nanoparticles (AuNPs) (C) known for their conductivity and surface easy modification properties, liposomes or virus-mimicking fusogenic vesicle (Vir-FVs) (D) for their biocompatibility and ability to encapsulate cargo, and innovative nanomaterials such as BP (black phosphorus) (E) for its unique surface-to-volume ratio, excellent biocompatibility and degradability properties. The microfluidic chip triggers fluorescence detection by loading liposomes or Vir-FVs fused with EVs to promote the binding of molecular beacons to the target (F). Surface-enhanced Raman scattering (SERS) has emerged as a potent technique for *in situ* EV nucleic acid detection, achieved by modifying Au@DTNB nanoparticles on the surface of EVs to act as Raman tags, thereby enabling SERS detection with high specificity and signal sensitivity (G). (Created with Figdraw.com).

Presently, there have been significant advancements in EV-based nucleic acid analysis techniques. Along with traditional PCR amplification, next-generation sequencing, microarray chip, and northern blotting, newer detection techniques such as fluorescence, electrochemical, colorimetric, SERS, SPR, and microfluidic biosensors have shown promising performance 11, 87, 88. However, these methodologies are constrained by several limitations, such as the necessity for a substantial sample size to ensure effective EV isolation, as well as labor-intensive and time-consuming procedures like EV lysis, RNA extraction, and cDNA synthesis. Moreover, RNA degradation and loss due to the lack of protection from the EV membrane are also concerns 16. Conversely, *in situ* detection strategies for EV nucleic acids have emerged as a solution, avoiding these limitations and improving diagnostic reliability. This section will discuss various *in situ* detection strategies reported thus far, including fluorescence assays based on different probe structures, microfluidic chips integrating fluorescence, and SERS assays (**Figure 3**).

### Fluorescence-based detection

Fluorescence-based analysis offers rapid speed, high sensitivity, excellent selectivity, and convenient operation, making it widely used in biology, medicine, environment, and food-related applications in recent years 89-91. The essential components for fluorescence detection include probes that hybridize with target molecules and active fluorescent groups attached to the probes. Researchers have optimized the probe's structure and enhanced fluorescence signal output efficiency to enable effective target analysis. Here, we summarize fluorescence detection strategies based on the probes' structural characteristics (**Table 1**).

#### Molecular beacon-based fluorescence detection

Molecular beacons (MBs) are labeled stem-loop oligonucleotide chains with fluorescent and quenching groups at the end. In their unbound state, MBs form a hairpin conformation where the fluorescent and quenching groups are close, leading to fluorescence quenching. When they hybridize with the target, the stem region of the MBs unfolds, increasing the distance between the fluorescent and quenching group, resulting in the restoration of fluorescence 92. MB-based fluorescence detection has been demonstrated for *in situ* detection of EV miRNAs. For example, Wu *et al.* developed a modularized DNAzyme-amplified two-stage cascaded hybridization chain reaction (CHCR-DNAzyme) circuit using multiple MBs. The output of the first hybridization chain reaction (HCR1) triggers the subsequent hybridization chain reaction (HCR2), producing numerous Mg^2+^-dependent DNAzyme branched nanowires for enhanced fluorescence detection. Through electroporation, their setup successfully detected miR-21 in oral squamous cell carcinoma cells, indicating potential for cancer diagnosis 93.

To overcome the heterogeneity of EVs and the limitation of a single biomarker and accurately reflect disease symptoms and stages, methods that simultaneously detect surface protein and nucleic acid biomarkers are needed. Cho *et al.* reported a multiplexed *in situ* EV assay platform using a CD63 antibody for EV capture combined with fluorescent-labeled MBs to detect miR-21 and miR-574-3p within EVs. This enables quantitative analysis of PCa cell-derived EV miRNAs and proteins at the single EV level 94. Additionally, He *et al.* constructed a Mg^2+^-dependent split DNAzyme probe (SDP) containing two divided DNAzyme fragments (D1 and D2) and an MB. Upon entering EVs, the SDP activates and cleaves the MB's stem-loop region in the presence of miR-21, generating fluorescence signals 95.

However, the efficiency of MB penetration into EVs alone is limited. Though electroporation or treatment with streptolysin O (SLO) can facilitate MB transport into EVs, it may disrupt the EV membrane and cause internal cargo leakage.

#### DNA nanostructure-based fluorescence detection

DNA nanostructures, such as nanowires, tetrahedrons, and cubes, have shown promise in cellular drug delivery and biosensors. These structures are programmable and stable, allowing them to penetrate biological membranes and protect substrates from interference effectively. They also confine molecular probes, reducing the risk of quenching and degradation 96-98. For example, DNA nanowires composed of complementary sequences are resistant to nuclease degradation and can be used for *in situ* detection of EV nucleic acids 99. Zhang *et al.* have developed a novel NgCHA nanoprobe using DNA nanowires assembled from two hairpins and a single-stranded DNA in combination with catalytic hairpin assembly (CHA), which enables direct detection of EV-miRNAs with high stability and penetration, showing great potential in EV-based disease management. Within the CHA process, two hairpins (H1, H2) are immobilized on the nanowire, and the miRNA target triggers the toehold strand displacement assembly of the two hairpins, thus recycling the miRNA and CHA products 100. DNA tetrahedrons, known for their endocytosis capabilities, have also been utilized in nanoprobe design 101. One study designed a fLIGHT nanoprobe with an integrated DNA tetrahedron and hairpin probe. The fLIGHT nanoprobe consists of three vertices with fluorescence donors, while the fourth vertex is located near the quencher of the hairpin probe. In the absence of the target, the three fluorescent groups can bind to the quenching group, resulting in the fluorescence turning off. When the target miRNA is present, it disrupts the stem-loop structure, leading to the quencher moving away from the vertices and activating the fluorescence. The fLIGHT nanoprobes can directly visualize miRNAs in EVs without damaging the EV membrane or extracting the EV cargo, creating a novel approach for *in situ* tracking of EV cargoes 102. In addition, DNA cubes have been utilized for *in situ* detection of EV nucleic acids. Chen *et al.* developed a DNA cube-based DDCA nano platform for the rapid, reliable, and sensitive detection of EV miRNA-21, showing potential in cancer screening and cellular communication studies. This approach utilizes a DNA nanocube and two hairpin DNAs (H1 and H2) within the EV. The target miRNA binds to H1, forming an intermediate structure, which then hybridizes with H2 to create an H1-H2, releasing the target miRNA for further cycles. This process brings the fluorescent groups of the two hairpins close together, enabling fluorescence resonance energy transfer (FRET) 103. Another study also showed that three-dimensional MBs based on DNA nanocages can effectively and non-destructively detect EV miRNAs *in situ* 104.

While DNA nanostructure-based probes enhance EV uptake efficiency and prevent cargo leakage, nanostructures that can assemble multiple probes for signal amplification to improve detection sensitivity are needed. Further understanding of the self-assembly mechanism and penetration process of nanostructures into the EV interior is also necessary.

#### Au nanoparticle-based fluorescence detection

Gold nanoparticles (AuNPs) have great potential in biomedicine due to their electrical conductivity, surface plasmon resonance, and accessible surface modification 105, 106. AuNPs-based nanoprobes with high loading capacity and efficient membrane penetration enable sensitive analysis of EVs through different amplification strategies. For example, Liu *et al.* developed a dual miRNA-activated, entropy-driven catalysis (EDC)-enhanced system for accurate detection of HCC cell-derived EV miR-21 and miR-122. This system consists of miRNA detection modules (SN), a reporter module (TA), and a signal amplification module (FA). Target EVs trigger a toehold-mediated strand displacement (TMSD) reaction, releasing the initiator (N) and initiating a cyclic reaction to generate an amplified fluorescent signal 107. Moreover, the combination of Au nanoflare and CHA amplification allows *in situ* and sensitive detection of EV miRNAs. Three different fluorescently labeled H1 hairpins (specifically binding miR-21, miR-122, and miR-375) ligated to AuNPs allow entry into the EVs directly. The fluorescence is activated upon recognition and binding of the target miRNAs by H1, and hairpin H2 subsequently initiates the hybridization chain reaction (CHA) to amplify the fluorescent signal response 108. Thermophoresis, induced by localized laser irradiation, derives the directional migration of fluorescently labeled EVs toward the center of the laser spot, leading to amplified fluorescence signals 109. Professor Sun's team implemented a thermophoretic sensor in nanoflare for *in situ* sensitive detection of breast cancer-derived EV miR-375. The miRNA reporter probe-modified AuNPs on nanoflares bind to the target miRNA in the EVs, generating a fluorescent signal. Subsequent thermophoretic enrichment of nanoflare-loaded EVs through localized laser heating enhances the fluorescence signal 110. Although AuNPs show promise for *in situ* EV detection, their penetration efficiency into EVs needs improvement. The size of particles directly influences the toxicity and biocompatibility of AuNPs. Ultrasmall AuNPs with diameters less than 10 nm have been found to exhibit superior cell penetration capabilities 111.

#### Liposome or Vir-FV -based fluorescence detection

Recent advancements in targeted drug delivery have been facilitated by employing liposomes and membrane vesicles, which exhibit outstanding biocompatibility 112, 113. Leveraging the membrane characteristics of EVs, researchers have employed membrane fusion strategies to deliver molecular probes enclosed in liposomes and virus-mimicking fusogenic vesicles (Vir-FVs) to enable *in situ* detection of nucleic acids. For instance, Liu *et al.* developed a sensitive and tethered cationic lipoplex nanoparticles (tCLN) biochip for identifying EV miRNAs in NSCLC patient serum. Within the tCLN biochip, cationic lipoplex nanoparticles with molecular fiducials are anchored on a gold-coated glass surface. Through electrostatic interactions, positively charged liposome nanoparticles and negatively charged EVs undergo fusion, followed by the hybridization of MBs in the liposomes with targets and the generation of fluorescent signals. This enables quantitative detection of EV miRNAs using TIRF microscopy 114. Another study utilized the innate RNase activity of bacterial Cas13a to directly measure EV miRNA in plasma by combining liposomes packaging CRISPR/Cas13a and reporter probes with EVs 115. However, the efficiency of membrane fusion mediated by these strategies requires improvement. Directing specific membrane fusions through DNA zipper hybridization enables rapid fusion kinetics. Researchers used liposomes and MBs to prepare probe-containing lipid vesicles and cholesterol-modified A/B′ double-stranded DNA (ZDC) through co-extrusion. At the same time, EV ZDC complementary to the liposomal ZDC was also prepared. Liposomes and EVs emit fluorescence upon hybridization of the probe to the target molecule through ZDC-mediated membrane fusion, enabling rapid detection of EV miRNAs for cancer diagnosis 116. Aside from liposome-mediated *in situ* EV detection, virus-mimicking fusogenic vesicles (Vir-FVs) can also facilitate efficient fusion with EVs. Gao *et al.* constructed Vir-FVs expressing hemagglutinin-neuraminidase (HN) protein and fusion (F) protein, allowing Vir-FVs directly target the salivary acid receptor on EVs and induce effective fusion. MBs enclosed in Vir-FVs can specifically target EV miRNAs, providing a promising strategy for cancer diagnosis and therapeutic monitoring 117. The studies mentioned above have demonstrated the possibility of using liposome- or Vir-FVs-based assays for *in situ* EV detection. However, it is essential to note that their instability could potentially lead to the premature release of the delivered MBs.

#### Novel nanomaterial-based fluorescence detection

In addition to AuNPs, various new materials have emerged in recent years for the development of biosensors, including quantum dots, graphene, hydrogels, metal oxide nanoparticles, carbon nanotubes, and upconversion nanoparticles. Black phosphorus (BP), a novel two-dimensional nanomaterial, possesses a honeycomb folded structure formed by strong intralayer P-P covalent bonds and weak interlayer van der Waals forces. It has a large surface-to-volume ratio, as well as good biocompatibility and degradability 118. The stability of BP can be enhanced by modifying it with Mn^2+^, which also promotes the adsorption of single-stranded DNA on the BP surface 119. Xia *et al.* recently developed a BP@Mn^2+^/DNA nanosensor by adsorbing miRNA fluorescent probes and EpCAM aptamers onto Mn^2+^-modified BP. This nanosensor enables direct detection of cancer-specific EV miRNAs and can easily penetrate the lipid bilayer membrane. Upon membrane penetration, the adsorbed DNA probe hybridizes with EV miR-21 and is released from the BP@Mn^2+^ surface, leading to the recovery of fluorescence signals 120. The development of the BP@Mn^2+^/DNA nanosensor provides a new approach for the rapid and efficient detection of *in situ* EV nucleic acids. Future advancements are expected to yield more sensitive and innovative 2D nanosensor platforms for cancer diagnosis.

### Microfluidic chips integrating fluorescence strategies

Microfluidics enables precise manipulation of fluids in small channels, offering high sensitivity, integration, and minimal sample and reagent consumption. It allows for the consolidation of multiple functions on a single chip, streamlining analysis and simplifying operational procedures 125. Currently, most of the microfluidic-based assay platforms incorporate fluorescence principles, with many designed for both EV isolation and nucleic acid detection 126, 127. However, only a limited number of these platforms have been documented to be utilized for *in situ* analysis of EV nucleic acids. Qian *et al.* introduced the isExoCD, a microfluidic chip based on agarose for EV concentration and *in situ* detection of EV miRNA. The chip utilizes capillary effect and agarose gel's permeability to concentrate the loaded mixture at one end of the microchannel, and any excess probes exit through the agarose gel nanopore. The isExoCD incorporates a CHA-based amplification strategy, enabling highly sensitive detection of EV miRNAs without complex elution steps or sample destruction 128.

Additionally, incorporating liposomes or Vir-FVs fusion with EVs has improved probe detection efficiency. For example, the mCLN-based assay with a micro-mixer biochip enables fast and sensitive detection of EV TTF-1 mRNA and miR-21 129. Another study developed a hydrogel microfluidic device that encapsulates liposomes containing probes, facilitating the release of the target gene as the hydrogel degrades and activating the CHA reaction for fluorescence signal amplification 130. Furthermore, Zhou *et al.* designed a 3D microfluidic chip with dual channels for the simultaneous detection of EV proteins and miRNAs. The chip recognizes tumor EV proteins using quantum dot-labeled antibodies (CD81, EphA2, and CA19-9) and detects EV miRNAs *in situ* by preparing Vir-FVs containing MBs fused with the EV 131.

Microfluidic-based *in situ* EV nucleic acid sensing platforms offer significant advantages in throughput, speed, and sample consumption. However, current detection methods heavily rely on liposomes or Vir-FVs for *in situ* analysis, highlighting the need to develop analytical strategies with improved permeability efficiency and non-destructive EV detection.

### SERS-based detection

Surface-enhanced Raman scattering (SERS) is a powerful technique that amplifies Raman signals near plasma nanostructures by attaching the analyte to its surface. It has been widely applied in food safety, environmental monitoring, and disease diagnosis due to its high sensitivity, signal specificity, and resistance to photobleaching 132. Although researchers have developed various SERS-based EV detection strategies for cancer diagnosis, only one report has been published on *in situ* EV analysis. Jiang *et al.* developed a Fe_3_O_4_@TiO_2_-based SERS sensing platform using LNA probe-modified Au@DTNB nanoparticles as tags. These tags were transferred to EVs through incubation and hybridization with target miRNAs, generating strong SERS signals. Additionally, Fe_3_O_4_@TiO_2_ nanoparticles were incorporated to bind the phosphate group of EVs, enabling Raman laser-based SERS detection. This SERS sensor demonstrated high sensitivity (LOD= 0.21 fM) and simplicity in directly quantifying EV miR-10b in PDAC-derived serum, providing a rapid and accurate method for EV-based cancer diagnosis 133. Further exploration of SERS strategies with high throughput and strong Raman signals is needed.

In summary, we present a comprehensive overview of the different strategies currently employed for *in situ* EV analysis, with fluorescence-based methods showing promising outcomes. However, probes enter EVs randomly, which may introduce interference from impurities in the sample, resulting in inaccurate target identification and potential false positive signals. Therefore, researchers must address background interference, develop sensitive and efficient probes, and facilitate EV translocation and target-specific recognition.

## Nucleic acid-based single EV assays

The heterogeneity of EVs brings significant challenges in achieving precise tumor diagnosis 134. Traditionally, EV analyses have often treated them as a homogeneous group, potentially masking the intricate biomarker details present in tumor-specific EVs and thus compromising the sensitivity and specificity of assays such as nanoparticle tracking analysis (NTA) and microfluidics. In contrast, single EV analysis, which focuses on the phenotype and characteristics of individual EV particles, offers a significant advantage in addressing this vesicular heterogeneity. This approach allows for a more nuanced understanding of the EV population, enhancing the potential for accurate biomarker detection and analysis.

**Figure 4:**
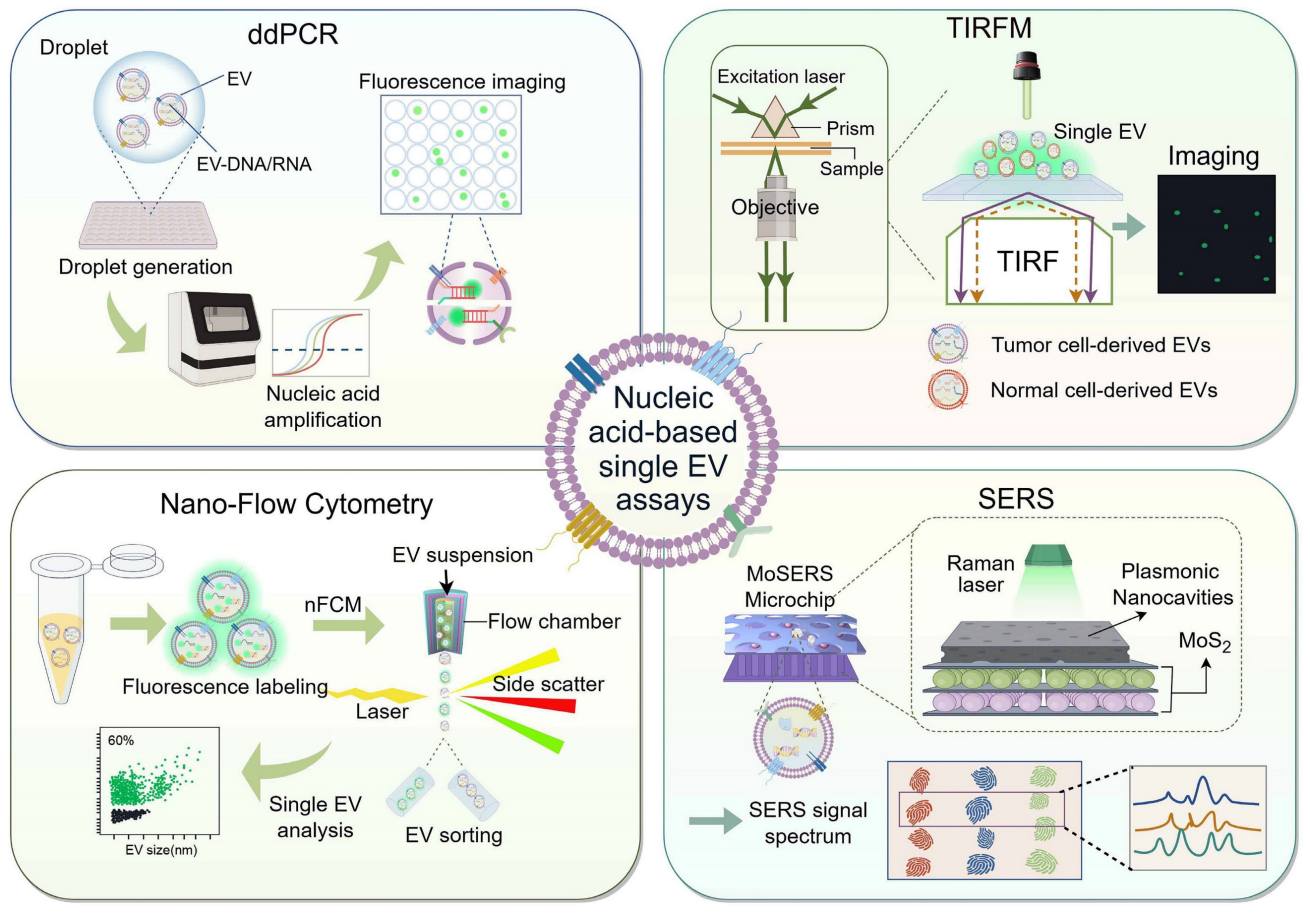
**Schematic representation of single EV analysis strategy for *in situ* nucleic acid detection.** Presently, nucleic acid-based single extracellular vesicle assays are classified into four categories: droplet digital PCR (ddPCR), total internal reflection fluorescence (TIRF) microscopy (TIRFM), Nano-flow cytometry, and surface-enhanced Raman scattering (SERS). In ddPCR systems, the EVs and the PCR reaction mixture are co-encapsulated within droplets, which are then evenly distributed across various reaction chambers to facilitate signal amplification (top left). TIRF microscopy elicits evanescent waves, which in turn excite fluorescent moieties within the EVs to generate fluorescent signals for subsequent imaging and quantitative assessment of the EVs (top right). Nano-flow cytometry can detect nanoparticles smaller than 200 nm, facilitating the capture and subsequent analysis of fluorescently labeled extracellular vesicles (EVs) to determine their concentration with the nano-flow cytometer (nFCM) (bottom left). The MoSERS microchip transforms the cargo information present on and within the EVs into a unique spectral fingerprint through the application of Raman laser illumination (bottom right). (Created with Figdraw.com).

Various methodologies are utilized for single EV analysis, including label-free analysis based on the physical characteristics of EVs and fluorescent labeling approaches rooted in their biological properties. Among these approaches, fluorescent labelling-based analyses can quantify protein and nucleic acid expression in single EVs, such as microdroplet digital PCR 135, digital ELISA 136, SERS 137, flow cytometry 138, and microscopic imaging 139. While there is some overlap between fluorescent labelling-based single EV analysis and *in situ* EV analysis, the current literature predominantly focuses on surface protein identification in single EV analysis. Furthermore, the integration of single EV analysis with *in situ* EV nucleic acid assays has not been extensively explored in existing studies. This section synthesizes recent advances in single EV analysis techniques through the lens of *in situ* EV nucleic acid assays (**Figure 4**).

### Droplet digital PCR

Droplet digital PCR (ddPCR) is a highly sensitive, specific, and quantitative polymerase chain reaction method that has become crucial for detecting rare targets. It involves dividing a sample into numerous microdroplets and distributing them evenly among different reaction units for amplification. The fluorescence signals produced by these reaction units are absolutely quantified by the statistical method of Poisson distribution 140. In RNA analysis within EVs, ddPCR typically requires extraction of EV RNA. Excitingly, ddPCR can be used to analyze RNA in individual EVs recently. Pasini *et al.* utilized the TaqMan-based ddPCR approach to analyze RNAs at the single EV level efficiently. By co-packing EV and PCR reaction solutions into microdroplets using the QX200™ Microdroplet Digital™ PCR System, they demonstrated a sensitivity exceeding 90% for EV-RNA-based EGFR mutation detection, with methods accuracy confirmed through Sanger sequencing 141. However, the mechanism by which the probe and PCR reaction solution enter the individual EV for target amplification remains unclear.

In addition to RNA, ddPCR can also detect DNA in single EVs. Recently, Jiao *et al.* developed a hydrogel-based droplet digital multiple displacement amplification (ddMDA) assay for precise analysis of DNA cargo in individual EVs 142. MDA is a highly effective whole genome amplification technique that uses Phi 29 DNA polymerase and randomly clustered primers to amplify even a single copy of DNA. In the ddMDA assay, hydrogel droplets containing individual EVs are generated using a vibrating sharp-tip capillary system and immobilized on a microfluidic chip. The cross-linked hydrogel traps the EVs while allowing the diffusion of small molecules and enzymes. By cleaving the EVs, the hydrogel enables the amplification of EV-DNA using ddMDA within a single droplet. This hydrogel-based ddMDA strategy not only enables the absolute quantification of DNA-containing EVs but also allows for the extraction of droplets containing fluorescent clusters for DNA sequencing. This provides a simple, sensitive, and powerful tool for early cancer detection and monitoring of treatment response.

### Total internal reflection fluorescence microscopy

Microscopic imaging methods, although capable of directly visualizing EVs through fluorescent labeling, are limited in their ability to differentiate between different EV subtypes and perform quantitative analysis. In recent years, total internal reflection fluorescence microscopy (TIRFM) has emerged as a promising technique for single EV analysis. By utilizing an evanescent wave produced by total internal reflection to excite fluorophores near the sample surface at a hundred-nanometer scale, TIRFM enables quantitative analysis of the emitted fluorescence signals 143. To detect EV RNA *in situ*, He *et al.* developed a TIRFM-based single vesicle imaging platform for quantitative and chemometric analysis of EV miRNA. This platform utilizes split DNAzyme probes that selectively attach to melanoma cell-derived EV miR-21, resulting in the emission of fluorescent signals captured by TIRFM. The TIRFM imaging assay allows for precise quantification of target miRNA at the single vesicle level and determination of the miRNA and EV stoichiometry. This platform is poised to become a universal and valuable tool for *in situ* quantification and stoichiometric analysis of disease-related EV miRNA biomarkers 144. Similarly, another study utilized TIRFM to simultaneously detect protein and mRNA/miRNA in a single EV, validated in a cohort of lung adenocarcinoma patients 145.

In addition, Zhang's lab proposed a single-EV and particle (siEVP) protein and RNA assay (^siEVP^PRA) assay to simultaneously detect protein and RNA biomarkers in multiple EV and lipoproteins subpopulations, revealing heterogeneity between vesicles and particles. The ^siEVP^PRA assay is manufactured from PRIMO optical modules containing circular micropattern arrays, and the signals of siEVP are visualized using TIRFM. The micropatterns include immobilized antibodies targeting the surface protein of EVP, enabling selective sorting of siEVP, while mRNAs and miRNAs are labeled with MBs. The ^siEVP^PRA assay has superior sensitivity compared to qRT-PCR (linear range of 10^6^-10^11^ particles/mL), and transcriptomic analysis reveals the heterogeneity of miRNAs in single EVs. Additionally, ^siEVP^PRA facilitates better differentiation between patients with glioblastoma (GBM) and healthy donors by detecting miRNA biomarkers in serum, presenting a novel approach for liquid biopsy and biomarker discovery 146.

### Nano-flow cytometry

Flow cytometry holds promise in EV analysis due to its capacity to assess data from individual cells. However, conventional flow cytometers have a detection limit for scattered light in the ranges from 300 to 500 nm, making it challenging to quantify and characterize nanoscale vesicles accurately. Although protein analysis of single EVs has been achieved by optimizing flow cytometers and incorporating fluorescence imaging, background interference and limited sensitivity remain issues 147, 148.

In recent years, nano-flow detectors have emerged as a potential solution for analyzing single EVs. These detectors overcome the limitations of conventional flow cytometry for particles smaller than 200 nm, enabling the characterization of individual particle sizes, distributions, concentrations, and biochemical properties with high sensitivity and throughput. This advancement herald new prospects in nanoscale flow detection technology 149. For example, Oliveira *et al.* employed a cell-penetrating peptide to deliver MBs into the EV and utilized a nano-flow assay to rapidly analyze the fluorescence signal of miRNA-451a, creating a new tool for MB-based RNA detection 150. However, the fast flow rate of the instrument and the need to detect faint fluorescent signals within a limited timeframe (average of 22 μs) pose challenges.

In terms of DNA detection, another study developed a nano-flow cytometer (nFCM) that can detect single EVs as small as 40 nm and single DNA fragments of 200 bp. This nFCM assay utilized SYTO 16 staining to analyze EV-DNA at the single vesicle level. The study identified DNA heterogeneity, including differences in DNA localization, quantity, and isoforms. Additionally, it revealed that cancer cells release a higher proportion of external DNA EVs, highlighting the potential of EV-DNA as a liquid biopsy strategy 151. This investigation provides direct experimental evidence for a comprehensive understanding of the relationship between DNA and EVs, in-depth offering a fresh insight into liquid biopsy strategies based on EV-DNA.

### Surface-enhanced Raman scattering

The rapid, non-destructive, and non-invasive nature of surface-enhanced Raman scattering (SERS) highlights its advantages in biosensing applications. Label-free SERS-based methods have the potential to convert the complete biochemical profile of EV into a unified spectral pattern or "fingerprint," making it an attractive tool for liquid biopsy applications 152, 153. However, the detection of biomarkers at the level of single EVs using SERS has not yet been achieved.

Recent studies have introduced a SERS-based microfluidic device called the MoSERS microchip, which incorporates embedded nanocavity arrays. These nanocavity arrays are equipped with MoS_2_ monolayers and layered plasma cavities, resulting in a significant enhancement of the electromagnetic field within the cavities. This allows for the acquisition of SERS spectra with single EV resolution without the need for additional biometric components. Additionally, the MoSERS microchip has been successfully used to characterize and analyze the cargo of single EVs, including the identification of mutations in glioma cells and circulating blood samples. These findings demonstrate the heterogeneity of EVs derived from glioblastoma cells and the potential of non-invasive liquid biopsies 154.

Single EV analysis is an emerging and fascinating research field that has the potential to revolutionize early cancer diagnosis. However, existing technologies often come with high costs and complicated procedures, and the purity of EV isolation is crucial for assay accuracy. It is essential to highlight that while our focus is on single EV-based nucleic acid analysis, the detection of multiple biomarkers can provide a more comprehensive understanding of the diversity of tumor EVs, particularly by examining proteins and nucleic acids.

## Machine learning for assisted *in situ* EV nucleic acid detection

**Figure 5:**
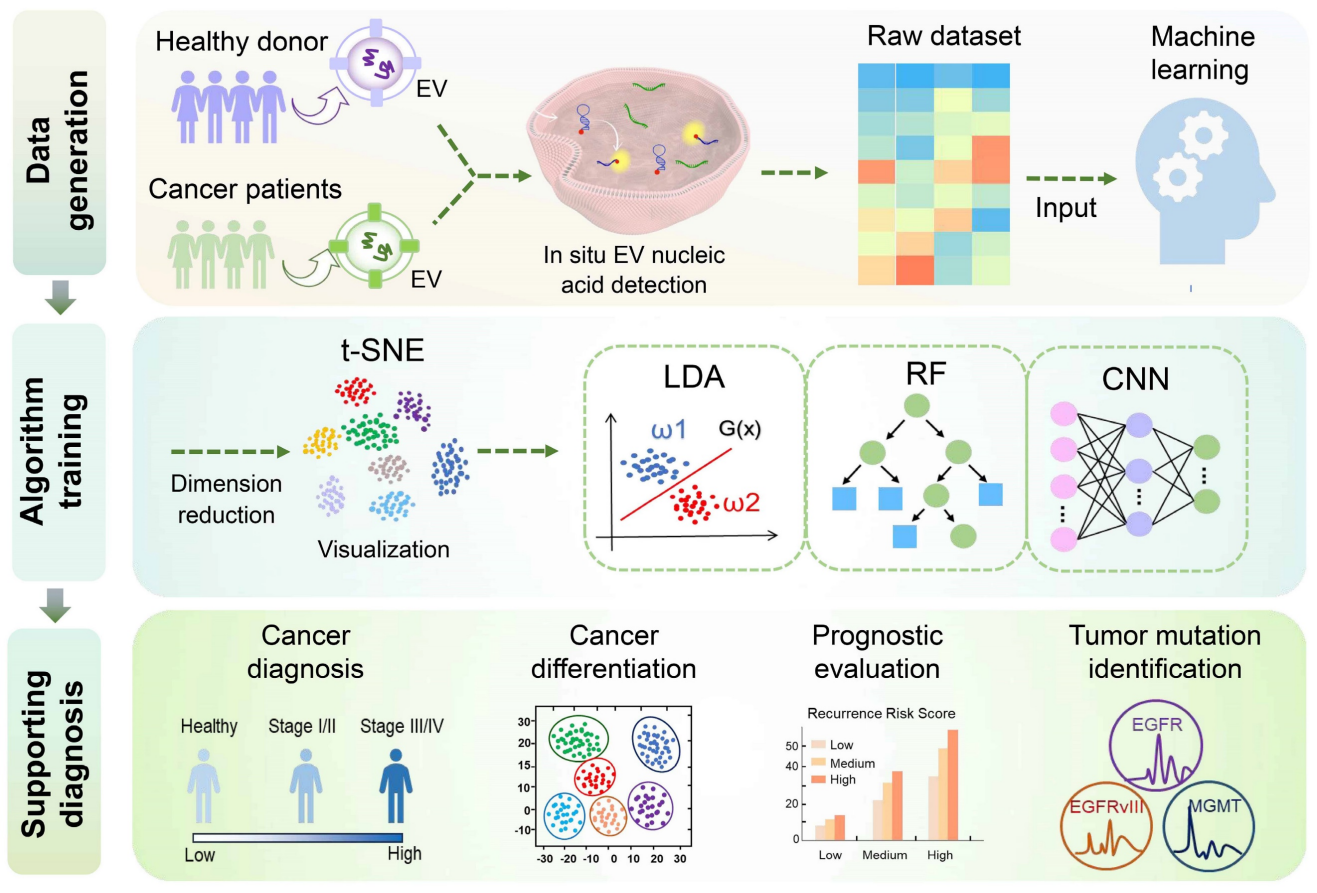
**Machine learning algorithms for assisted *in situ* EV nucleic acid analysis employed in liquid biopsies.** The incorporation of four machine learning algorithms, including linear discriminant analysis (LDA), random forest (RF), convolutional neural network (CNN), and t-distributed stochastic neighbor embedding (t-SNE), enhances the procedures involved in tumor diagnosis, differentiation, prognostic evaluation, and mutation identification utilizing the *in situ* EV nucleic acid analysis platform. Specifically, t-SNE enables visualization by reducing the dimensionality of raw data, and subsequently integrates with other algorithms to aid in distinguishing data characteristics, thereby enhancing the diagnostic efficacy of the model.

In recent years, machine learning (ML) has emerged as a promising tool for enhancing disease diagnosis, cancer classification, survival prediction, and treatment decision-making, leading to more reliable and accurate disease-specific diagnostic systems 155, 156. Several ML algorithms have been extensively employed in biomedicine, including principal component analysis (PCA), t-distributed stochastic neighbor embedding (t-SNE), linear discriminant analysis (LDA), support vector machine (SVM), random forest (RF), K-nearest neighbors (KNN), convolutional neural network (CNN), decision tree, logistic regression, and various others. In this context, we provide an overview of some widely adopted machine learning algorithms currently utilized for *in situ* EV detection (**Figure 5**).

### Linear discriminant analysis

LDA is a classical statistical machine learning algorithm used for classifying or distinguishing between different event features by identifying linear combinations of these features 157. It has been applied successfully in cancer classification, as demonstrated in a study involving 64 cases of five cancer types, including breast cancer, lung cancer (LC), hepatocellular carcinoma (HCC), colorectal cancer (CRC), and cervical cancer (CC). A nanoflare & CHA-based sensing platform integrated with the LDA algorithm can quickly and accurately differentiate five types of cancers in a non-invasive manner. Patients diagnosed with the five types of cancers exhibited minimal overlap in the LDA graph, achieving an overall accuracy of 99% 108. Moreover, Zhou *et al.* developed a 3D microfluidic chip that integrates two channels for simultaneous detection of EV proteins and miRNAs. By applying LDA analysis to evaluate the expression levels of EV biomarkers, the chip successfully differentiated patients with advanced pancreatic cancer (PC) from those with early-stage PC and benign controls. The classification results demonstrated a remarkable overall accuracy of 100% for cancer diagnosis and clinical staging, with individual EV biomarkers showing error rates ranging from 35% to 44% 131. In another exciting development, Ray *et al.* developed the SORTER assay, which detects a specific 6-miRNA signature in EVs derived from PCa. Validated using LDA, the assay exhibited 100% sensitivity, specificity, and accuracy in both training and validation cohorts, enabling the discrimination of PCa from benign prostatic hyperplasia patients. Interestingly, the identified PCa signature by SORTER was found to be unrelated to serum PSA levels 158.

### Random forest

RF algorithm combines multiple weak classifiers to achieve high accuracy and robust generalization performance through voting or averaging 159. It has been shown to predict tumor recurrence risk and contribute to cancer classification effectively. For instance, Zhang *et al.* used RF in conjunction with EV-miRNA signatures (miR-1246, miR-375, miR-221, miR-21) detected by the NgCHA nanoprobe assay to evaluate the recurrence risk in breast cancer patients and guide personalized treatment strategies. The RF-based risk assessment achieved an overall accuracy of 87% in the training cohort and 82% in the validation cohort, with EV-miR-1246 exhibiting significant influence. The model also accurately differentiated between different tumor types, with optimal accuracy observed in identifying breast and lung cancers. In comparison to other machine learning algorithms such as NN, SVM, and LDA, RF outperformed with a classification diagnosis accuracy of 58% in the validation cohort 100.

### Convolutional neural network

CNN is an advanced deep-learning algorithm that specializes in image analysis, a subfield of machine learning known for its robust auto-learning capabilities, making it particularly advantageous for analyzing intricate samples 160, 161. Jalali *et al.* used a CNN model to train the spectral fingerprint output from their MoSERS microarrays for distinguishing glioblastoma mutations. By collecting 946 SERS fingerprints of glioma cell EVs and dividing them into a training dataset (70% of the total data) and a test dataset (30% of the total data), the findings showed that CNN accurately classified individual EV spectra of different mutation types, achieving an overall classification accuracy of 89.3%. Moreover, CNN achieved an accuracy of 0.85 in the classification of circulating blood EVs containing various glioblastoma variants (EGFR amplification, EGFRvIII, and MGMT methylation) while also achieving an AUC value of 0.91 in discriminating between patients with GBM with genetic variants and healthy donors. Additionally, the MoSERS validation using binary CNN algorithms for EV segmentation showcased the predictive efficacy in identifying GBM variants (AUC=0.89), enhancing diagnostic precision of GBM mutations and presenting a promising analytical approach 154.

### T-distributed stochastic neighbor embedding

t-SNE stands out as a cutting-edge machine learning algorithm designed for dimensionality reduction. This method effectively transforms high-dimensional datasets into two-dimensional or three-dimensional representations for visualization, exhibiting superior dimensionality reduction compared to PCA 162. While the t-SNE algorithm excels at preserving the local structures with complex data sets but lacks predictive capabilities, it is often combined with complementary algorithms to enhance model performance. For example, the collaborative use of t-SNE and RF algorithms has demonstrated notable efficacy in distinguishing between various types of cancer. Zhang *et al.* prioritized the use of the t-SNE algorithm to map the properties of EV-miRNA panels in the training cohort to a two-dimensional plane for visualization in verifying the application potential of their constructed NgCHA *in situ* detection platform. Subsequently, the RF algorithm was utilized to differentiate the EV-miRNA features for identifying breast, lung, liver, gastric, and colorectal cancers 100. In addition, the t-SNE enables data classification post-dimensionality reduction. In the context of *in situ* detection of EV biomarkers utilizing the SORTER platform, researchers observed no significant correlation among multiple miRNAs associated with distinct EV subgroups. Therefore, they applied the t-SNE to distinguish between prostate cancer and benign prostatic hyperplasia across different EV subtypes and employed LDA to identify EV-miRNA profiles to improve the diagnostic performance of the platform 158.

The application of machine learning in EV liquid biopsy has opened up a new chapter in cancer diagnosis assistance, particularly in addressing the heterogeneity of EVs for cancer classification. In addition to the four ML algorithms described above, SVM, KNN, and other deep learning algorithms are expected to be utilized in the future for assisted diagnosis relying on *in situ* EV nucleic acid analysis. It is important to note that machine learning models require sufficient data for effective training. Limited clinical samples in current studies may reduce the model's capacity and distort the predicted outcomes. The choice of interpretable algorithms to help physicians understand the decision-making process also needs to be considered. Therefore, further improvements are needed to enhance the accuracy of these models.

## Application of *in situ* EV nucleic acid detection in liquid biopsy

**Figure 6:**
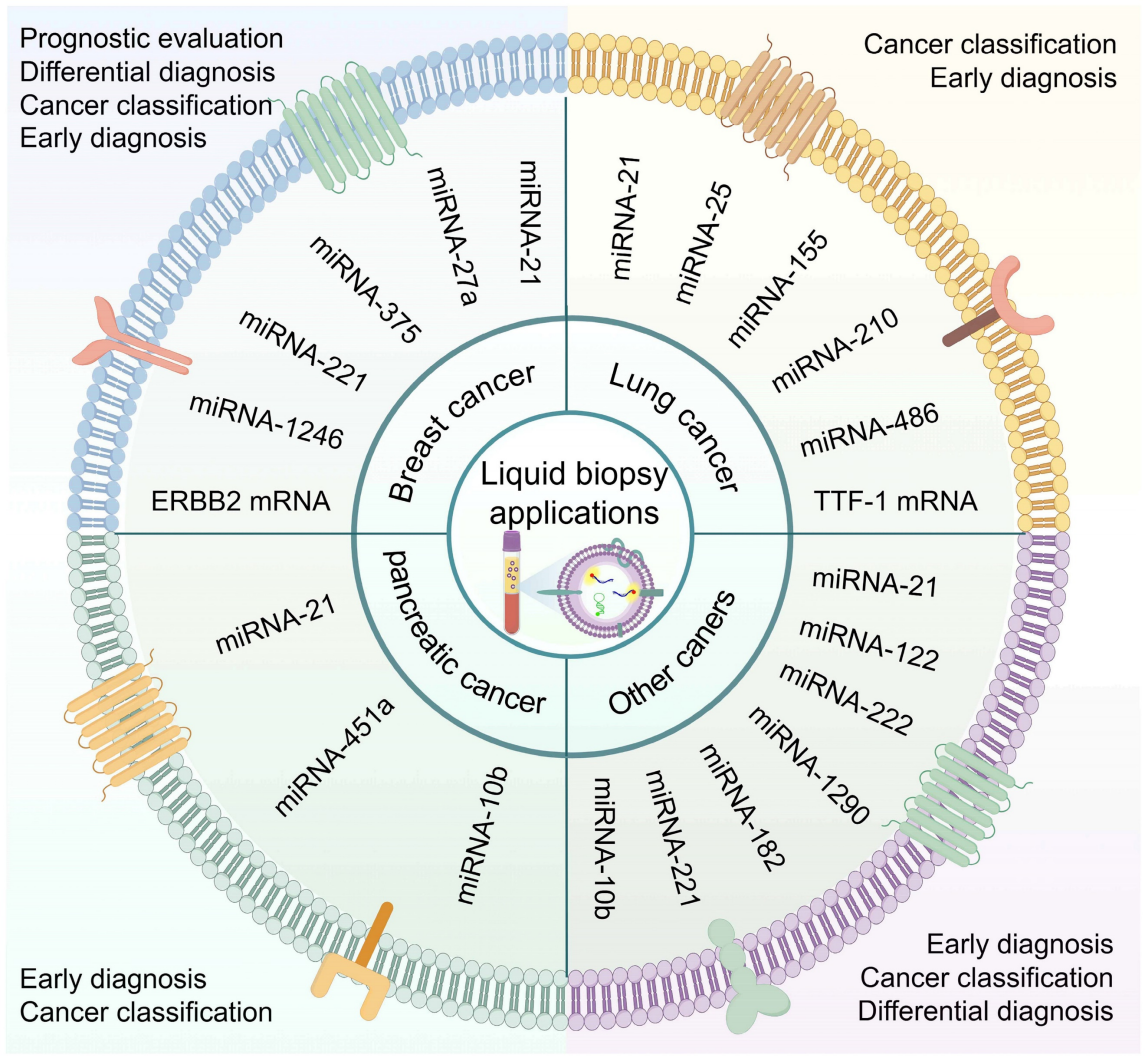
** Application of *in situ* EV nucleic acid detection in liquid biopsies.** *In situ* detection of EV nucleic acids shows promising prospects in early diagnosis, differential diagnosis, cancer classification, and prognostic evaluation in cancer. These assays have been validated across various types of cancer, including breast cancer, lung cancer, pancreatic cancer, and other cancers (hepatocellular carcinoma, colorectal cancer, prostate cancer), with EV miRNAs derived from EVs in circulating blood serving as the primary biomarkers. (Created with Figdraw.com).

EV-based liquid biopsies have made significant progress, with certain biomarkers advancing to clinical trials 163-165. However, detecting these biomarkers is often time-consuming, labor-intensive, and expensive. The development of *in situ* EV nucleic acid analysis technology has gained attention for its simplicity, speed, and affordability. Here, we illustrate the application of *in situ* EV nucleic acid analysis methods in liquid biopsies (**Figure 6**) and summarize their diagnostic performance in different types of cancer (**Table 2**).

### Breast cancer

Globally, early detection and treatment of breast cancer (BC) are crucial in reducing morbidity and mortality rates among women 1. Research has identified miR-21 and miR-375 as up-regulation biomarkers in various cancers, including BC, with implications for tumor progression and prognosis 167-171. *In situ* EV biomarker identification in BC has primarily focused on miR-21, followed by miR-375. For instance, Liu *et al.* developed a DNA cube-based DDCA nano platform capable of sensitive *in situ* detection of EV miR-21 in BC within 30 min. Clinical validation demonstrated higher FRET signals in BC patients compared to healthy individuals, as well as in advanced-stage patients compared to early-stage patients. This suggests the potential application of DDCA for early diagnosis of BC 103. In estrogen receptor (ER)-positive BC, the most prevalent subtype with a better prognosis, miR-375 plays a regulatory role. Zhao *et al.* reported the use of a thermophoretic sensor (TSN) utilizing nanoflares detecting breast EV miRNA. TSN achieved high sensitivity, with the ability to analyze miRNAs from small serum samples. The clinical application showed that EV miR-375 exhibited 90% accuracy in distinguishing between ER-positive BC patients and healthy donors, with an impressive AUC value of 0.96. Moreover, EV miR-375 demonstrated good sensitivity, specificity, accuracy, and AUC value of 0.94 for early-stage (stage I and II) detection of ER-positive BC, providing a potential tool for improving BC diagnosis 110. Additionally, liposome-based MFS-CRISPR platform, DNA-functionalized AuNPs probes, and multi-branched localized catalytic hairpin assembly (MLCHA) probes have shown promising diagnostic capabilities in discriminating BC patients from healthy donors 115, 123, 166.

Individual biomarker assays often lack accuracy and sensitivity. However, combining multiple biomarkers has significant advantages in precise cancer classification. Wang *et al.* developed a Y-scaffold probe that can detect multiple EV miRNAs (miR-21, miR-375, miR-27a) simultaneously *in situ* using competitive strand replacement. This approach showed higher miRNA fluorescence intensity in BC serum EVs compared to healthy controls, effectively distinguishing BC patients from healthy donors 121. Recently, NgCHA nanoprobes based on DNA nanowire-guided catalyzed hairpin assembly have also been used for BC diagnosis and recurrence risk assessment. Zhang *et al.* employed a panel of 4-EV-miRNAs (miR-221, miR-375, miR-1246, and miR-21) as potential biomarkers for NgCHA detection, and analysis of 36 clinical samples revealed significant variations in the expression levels of these four EV-miRNAs among patients. Discriminating BC from healthy donors, the combination of these four EV-miRNAs demonstrated the highest accuracy with an AUC of 0.945, 95% sensitivity, and 95% specificity in the early stages of BC. Moreover, EV-miRNA-1246 played a crucial role in recurrence risk assessment modeling 100.

### Lung cancer

Liquid biopsy plays a crucial role in the diagnosis and management of lung cancer (LC), which is a prevalent malignancy 172. One effective approach is utilizing DNA tetrahedrons as nanocarriers to transport hairpin probes into EVs. Chen *et al.* employed this method, known as fLIGHT, to detect EV miR-21. The nanoprobe demonstrated comparable performance to RT-qPCR in differentiating non-small cell lung cancer (NSCLC) patients from healthy individuals. The fLIGHT assay exhibited a strong correlation with RT-qPCR, suggesting its suitability for EV miRNA quantification 102. In addition, microfluidic-based biochips offer fast and sensitive detection of EV RNA. In one study, a microfluidic cationic lipid complex nanoparticle (mCLN)-based assay successfully detected miR-21 and TTF-1 mRNA in serum EVs, effectively distinguishing NSCLC patients from normal controls. Compared to the conventional qRT-PCR, the mCLN assay required a smaller sample volume (30ul vs. 100ul) and significantly reduced the processing time (10 minutes vs. 4 hours), demonstrating superior diagnostic accuracy in NSCLC 129. Moreover, a study utilizing a tCLN biochip validated the diagnostic value of five miRNAs (miR-21, miR-25, miR-155, miR-210, miR-486) in serum EVs from both early-and late-stage NSCLC patients. The tCLN assay exhibited excellent diagnostic performance when utilizing the combined panel of five miRNA in differentiating normal controls from all NSCLC patients. Notably, the tCLN assay showed absolute sensitivity and specificity in differentiating early-stage NSCLC patients from normal controls, highlighting its potential as a liquid biopsy assay for early NSCLC detection 114.

### Pancreatic cancer

MiR-10b is a commonly utilized diagnostic and prognostic biomarker for pancreatic ductal adenocarcinoma (PDAC) detected in EVs 173. Researchers focused on validating miR-10b as a target for *in situ* EV assays. Jiang *et al.* developed a SERS-based sensing strategy, incorporating an LNA-Au@DTNB label into serum and enriching EVs using Fe_3_O_4_@TiO_2_. The Raman assay demonstrated significantly higher miRNA-10b signals in the sera of PDAC patients, with an AUC of 0.996 for distinguishing PDAC from healthy controls. This was highly correlated (0.996) with qRT-PCR results 133. In addition, a comprehensive cancer diagnostic platform was designed to analyze multiple biomarkers at the single EV level by using a 3D microfluidic device, six EV markers (CD81, EphA2, CA199, miR-451a, miR-21, miR-10b) were simultaneously identified in 40 plasma specimens. The combined expression of CD81 (EphA2, miRNA-451a, miRNA-21, miRNA-10b) showed the best diagnostic value (AUC=1). Distinctly, EphA2, miRNA-451a, and miRNA-21 profiles could differentiate between healthy controls and early (stage I/II) pancreatic cancer patients. Moreover, miR-451a and miR-10b signatures were effective in discriminating between early-stage (I/II) and advanced-stage (III/IV) pancreatic cancer, indicating the strong potential of this platform for early cancer diagnosis 131.

### Other cancer (Hepatocellular carcinoma, Colorectal cancer, Prostate cancer)

In addition to the mentioned cancer types, *in situ* detection techniques have been used to diagnose other malignancies. For example, in hepatocellular carcinoma (HCC), an entropy-driven catalysis (EDC)-enhanced DNA Logical device based on AuNPs was employed to detect serum EV miR-21 and miR-122. Simultaneous detection of these two EV miRNAs effectively distinguished HCC patients from healthy individuals with an accuracy rate of 93.3% 107. In colorectal cancer (CRC), Xia *et al.* employed a BP@Mn^2+^/DNA nanosensor capable of differentiating EV miR-21 in plasma samples of CRC patients from healthy individuals. The sensor integrated an EpCAM aptamer to recognize CRC-specific EVs 120. Additionally, Lei *et al.* designed a SORTER assay for identifying tumor-derived EVs and analyzing miRNAs to improve diagnostic accuracy in prostate cancer (PCa). By detecting six specific miRNAs (miR-222, miR-1290, miR-182, miR-21, miR-221, and miR-10b) in EVs, SORTER achieved a flawless discrimination between PCa and benign prostatic hyperplasia (BPH) with an impressive accuracy rate of 100%. Moreover, it achieved a diagnostic accuracy of 90.6% in distinguishing metastatic from non-metastatic PCa, demonstrating the potential of miRNA-based liquid biopsy in clinical settings 158.

In conclusion, *in situ* EV analysis shows promise for early cancer diagnosis, cancer classification, differential diagnosis, and predicting recurrence risks. However, most detection methodologies primarily focus on established miRNAs as target markers, often using breast cancer models and overlooking the exploration of novel biomarkers in various circulating body fluids. Larger clinical sample sizes are needed to ensure the reliability of the results. Looking ahead, these detection platforms have the potential to expand their applications, including disease screening, prognosis determination, and monitoring treatment responses in the future.

## Challenges and perspectives

EVs have gained significant attention in liquid biopsy due to their abundance, stability, and diverse information reflecting disease progression in bodily fluids. These vesicles carry various biomarkers, with RNA-based molecules, particularly miRNAs, playing a crucial role. miRNAs, enriched in EVs and characterized by short sequences, show promise in early cancer diagnosis, prognostic assessment, and progress monitoring. Although other biomarkers have shown improved diagnostic capabilities, the utilization of DNA, mRNA, and lncRNA is less common due to EV volume limitation. CircRNAs, known for their superior stability and tissue specificity, are highly abundant in EVs, making them a potential focus for liquid biopsy strategies 73. Despite these advancements, clinical implementation of EV biomarkers still faces challenges, such as the lack of validated clinical samples, suboptimal diagnostic sensitivity and specificity, impurities in EV isolation, and limited sensitivity and reproducibility of detection methodologies. Most of the EV biomarkers studied so far have been identified in sEVs or exosomes present in different circulating body fluids. However, nucleic acid biomarkers derived from microvesicles, apoptotic vesicles, and platelet vesicles have not yet been detected *in situ*.

Numerous methodologies for EV nucleic acid detection have been extensively studied. However, these methods require demanding procedures involving EV lysis and nucleic acid extraction. Therefore, our attention is directed towards approaches for *in situ* EV nucleic acid analysis and adjunctive diagnostic strategies that combine single EV analysis and machine learning, particularly focusing on their prospective utility in liquid biopsy. These innovative tools have significant potential in contemporary liquid biopsies for rapid and direct acquisition of information on EV biomarkers for tumor diagnosis, classification, and prognostic evaluation. The goal of *in situ* EV nucleic acid analysis is to develop sensitive, precise, stable, and high-throughput assay platforms integrated with single EV analysis to identify tumor-specific EVs and implement them effectively in liquid biopsies. Nevertheless, several challenges need to be addressed in the future. The following insights emphasize the unaddressed needs of these strategies in the context of diagnostic applications for *in situ* nucleic acid analysis of EVs.

1. The advancement of fluorescence-based *in situ* EV nucleic acid detection has made rapid progress. DNA nanostructured and AuNPs probes can directly penetrate EVs without requiring membrane treatment. However, further refinement is needed to enhance EV penetration efficiency and increase the stability of the assembled probes. Moreover, fluorescence detection is prone to interference from impurities. So, it is advisable to minimize self-induced fluorescence by using ratiometric fluorescence detection. Furthermore, the effectiveness of this approach should be confirmed by employing conventional detection techniques.

2. *In situ* EV nucleic acid detection requires high sensitivity. Current signal amplification strategies, such as CHA and CHR, involve the use of multiple probes. Therefore, it is imperative to explore methods that enable efficient integration of more probes into EVs while facilitating EV translocation. Additionally, future advancements are expected to introduce novel nanomaterials for application in *in situ* detection.

3. Microfluidic chips integrating fluorescence strategies demonstrate excellent performance in terms of high throughput, rapid detection, and minimal sample consumption. However, there is a need to enhance their sensitivity and stability. In addition to the promising SERS assays, it is expected that *in situ* analysis strategies for EV nucleic acids employing electrochemical, colorimetric, and surface plasmon resonance (SPR) methods will be developed and applied in liquid biopsies.

4. Single EV analysis is valuable for comprehending cancer heterogeneity and pinpointing diagnostic biomarkers. However, current platforms for directly analyzing nucleic acids at the single EV level, such as digital PCR, nano-flow cytometry, and TIRFM, are expensive and face challenges in clinical adoption. Microfluidic devices based on SERS exhibit promising potential for application. It is crucial to characterize multiple biomarkers at the single EV level to improve diagnostic accuracy.

5. The machine learning methods (LDA, RF, CNN, t-SNE) employed for assisting *in situ* EV nucleic acid detection demonstrate excellent analytical capabilities in discriminating between cancer and healthy donors and categorizing different cancer types, thereby enhancing diagnostic accuracy. However, the small sample size of the proposed detection method may pose a challenge and require algorithm optimization or integration with other algorithms for more realistic outcomes. Training before validation is advisable to enhance the algorithm's learning capacity and assess accuracy effectively. Additionally, simultaneous detection of multiple biomarkers combined with machine learning enables fast and accurate cancer diagnosis. Integrating microfluidic-based high-throughput methods with machine learning presents a promising strategy.

6. *In situ* EV nucleic acid assays promise to advance liquid biopsy technology, but factors such as sample volume and assay time hinder clinical implementation. Techniques to identify various biomarkers and the integration of multiple biomarkers can facilitate the precise characterization of tumor-derived EVs for accurate diagnosis. Additionally, the *in situ* detection of DNA mutation and novel biomarkers (e.g., circRNA, lncRNA) will offer potential for comprehensive integration into future liquid biopsy applications.

In conclusion, we emphasize the importance of *in situ* EV nucleic acid-based detection techniques in tumor liquid biopsy. Integration of solutions for single EV analysis and machine learning is expected to enhance *in situ* detection methods, enabling swift and effective precision medicine practices.

## Figures and Tables

**Table 1:** Performance comparison of four popular fluorescence strategies.

Methods	Probe Stability	Detection flux	Penetration efficiency	EV integrity	Ref.
Molecular beacon-based fluorescence detection	Stable at room temperature	Multiple molecular beacons can be transported to EVs	Requires membrane treatment to facilitate penetration	Electroporation and SLO treatment destroy membrane structure	93, 94, 121
DNA nanostructure-based fluorescence detection	Inefficient stabilization of self-assembly	Confined space restricts the carrying of probes	Rigidity and hardness of nanostructures provide better permeation efficiency	Have better integrity	101, 103, 122
Au nanoparticle-based fluorescence detection	Related to the way of probe modification onto the surface of AuNPs	Surface of AuNP can be modified with multiple probes	Related to the size of AuNPs	Have better integrity	108, 123, 124
Liposome or Vir-FV -based fluorescence detection	Poor stability, needs to be ready to use	Generally, only one type of probe is loaded	Instability of liposomes may affect their fusion efficiency with EV membranes	Membrane is fused	112, 114, 115

EV: extracellular vesicle; SLO: streptolysin O; Vir-FV: virus-mimicking fusogenic vesicle.

**Table 2:** The significance of *in situ* extracellular vesicle nucleic acid analysis in liquid biopsy of cancer.

Biomarker	Sample	Test Methods	Cases of tumors	Linear range	LOD	Time	AUC	Sample volume	Ref.
miR-21, miR-122, miR-375	BC, LC, HCC, CRC, CC derived plasma	Fluorescence	64	0.0195 fM-19.5 pM	5 aM	2h	0.429, 0.561, 0.556 (combined= 0.944)	500ul plasma	108
miRNA-21	BC derived serum	Fluorescence	22	2.5×10^5^-1.5×10^7^ particles/ul	9.8×10^4^ particles/ul	30min	/	/	103
miR-21, miR-122	HCC derived serum	Fluorescence	15	2.4×10^5^-1.7×10^6^ particles/ul	1.2×10^5^ particles/ul	3.5h	0.92	/	107
miR-375	BC derived serum	Fluorescence	15	/	6.53×10^3^ particles/ul	3h	0.92	/	123
miR-375, miR-1246, miR-221, miR-21	BC derived plasma	Fluorescence	21	1 pM-50 nM	0.8 pM	30 min	0.756, 0.894, 0.810, 0.875 (combined=0.965)	1ml plasma	100
miRNA-27a	MCF-7-tumor-bearing mice derived plasma	Fluorescence	10	0.76×10^6^-15.24×10^6^ particles/ul	1.9×10^4^ particles/ul	3.5h	0.95	5ul plasma	166
miR-21, miR-25, miR-155, miR-210, miR-486	NSCLC derived serum	Fluorescence	64	/	/	2.5h	0.97	60ul resum	114
miR-21, miR-27a, miR-375	BC derived serum	Fluorescence	3	/	0.116 μg/mL, 125 μg/mL, 0.287 μg/mL	30min	/	/	121
miR-21	BC derived serum	Fluorescence	4	1.25-200 nM	0.5 nM	30min	/	5ml serum	116
miR-21	BC derived plasma	Fluorescence	10	10^1^-10^5^ particles/ul	1.2 particles/ul	3.75h	0.84	500ul plasma	115
miR-21	Melanoma, CC, BC derived serum	Fluorescence	3	3×10^4^-10^7^ particles/ μL	378 copies/μL	1h	/	/	144
miR-21- 5p	NSCLC derived serum	Fluorescence	10	1×10^-12^-10×10^-9^ M	45.4×10^-15^ M	20min	/	/	102
miR-21	BC derived serum	Fluorescence	5	/	/	2h	/	/	117
miR-21	CRC derived plasma	Fluorescence	10	5×10^2^-2×10^4^ particles/μl	1.86×10^2^ particles/ul	30min	/	200ul plasma	120
miR-375	BC derived serum	Fluorescence	17	0.17-170 fM	0.36 fM	2h	0.96	0.5ul serum	110
miR-451a, miR-21, miR-10b	PC derived plasma	Microfluidic chip and fluorescence	30	/	0.71×10^-9^ M, 1.74×10^-9^ M, 1.28×10^-9^ M	1h	0.930, 0.939, 0.875	2ul plasma	131
miR-222, miR-1290, miR-182, miR-21, miR-221, miR-10b	PCa derived plasma	Microfluidic chip and fluorescence	47	/	0.8×10^5^ particles/ul	2h	combined=1	0.2ul plasma	158
miRNA-10b	PDAC derived serum	SERS	15	0.33 fM-1.65 pM	0.21 fM	4h	0.996	4ul serum	133
miR-21, TTF-1 mRNA	NSCLC derived serum	Microfluidic chip and fluorescence	10	8×10^4^-8×10^7^ particles/ul	3.71×10^6^ particles/ul, 2.06×10^6^ particles/ul	10min	/	30ul serum	129
ERBB2	MCF-7-tumor-bearing mice derived plasma	Microfluidic chip and fluorescence	8	100 fM-1 μM	58.3 fM	2h	/	400ul plasma	130

BC: breast cancer; LC: lung cancer; HCC: hepatocellular carcinoma; CRC: colorectal cancer; CC: cervical carcinoma; NSCLC: non-small cell lung cancer; PC: pancreatic cancer; PCa: prostate cancer; PDAC: pancreatic ductal adenocarcinoma.

## Abbreviations

EV: extracellular vesicle
SERS: surface-enhanced Raman scattering
SPR: surface plasmon resonance
M2ф: M2-like macrophages
PDAC: pancreatic ductal adenocarcinoma
CAF: Cancer-associated fibroblast
Treg: regulatory T cell
DC: Dendritic cell
M1ф: M1-like macrophages
CRC: colorectal cancer
GC: gastric cancer
HCC: hepatocellular carcinoma
mtDNA: mitochondrial DNA
NSCLC: non-small cell lung cancer
EC: endometrial cancer
SCLC: small-cell lung cancer
PCa: prostate cancer
MB: molecular beacon
SLO: streptolysin O
AuNP: gold nanoparticle
Vir-FV: virus-mimicking fusogenic vesicle
BP: black phosphorus
ddPCR: droplet digital PCR
TIRFM: total internal reflection fluorescence microscopy
GBM: glioblastoma
LDA: linear discriminant analysis
RF: random forest
CNN: convolutional neural network
t-SNE: t-distributed stochastic neighbor embedding
BC: breast cancer
LC: lung cancer
